# The role of telomerase reverse transcriptase (TERT) promoter mutations in prognosis in bladder cancer

**DOI:** 10.1080/21655979.2021.1915725

**Published:** 2021-05-02

**Authors:** Song Wan, Xuan Liu, Wei Hua, Ming Xi, Yulin Zhou, Yueping Wan

**Affiliations:** Department of Urology, Huadu District People’s Hospital of Guangzhou, Guangzhou, China

**Keywords:** Bladder cancer, tert promoter mutations, prognosis

## Abstract

Telomerase reverse transcriptase (TERT) promoter mutations have been recognized as a common genetic event in bladder cancer (BC). Many studies have found the high TERT promoter mutations’ prevalence in BC recurrence patients which may make the TERT promoter mutations become a potential prognosis prediction of BC. We performed a systematic search in Embase, PubMed, and Web of Science in January 2021. The aspects of evaluation, methods, validation, and results were used to evaluate the included studies’ quality. We reviewed two of the most common mutations in types of TC, C288T and C250T and their relationship with prognosis of BC. Eight studies contained 1382 cases were enrolled in our study. The percentage of TERT promoter mutations in these cases was 62.5%. A statistically significant association was detected between TERT promoter mutation and recurrence (HR: 2.03, 95% CI: 1.53–2.68, p < 0.001). However, TERT promoter mutation was not significant associated with overall survival (HR: 1.077, 95% CI: 0.674–1.718, p = 0.757). No significant heterogeneities were observed (I^2^ = 47.5%, P = 0.064; I^2^ = 58.7%, p = 0.120, respectively). Bladder cancer patients with TERT promoter mutations take a higher risk of recurrence. TERT promoter mutations may become a potential prediction factor for bladder cancer recurrence.

## GRAPHICAL ABSTRACT

**Figure:**
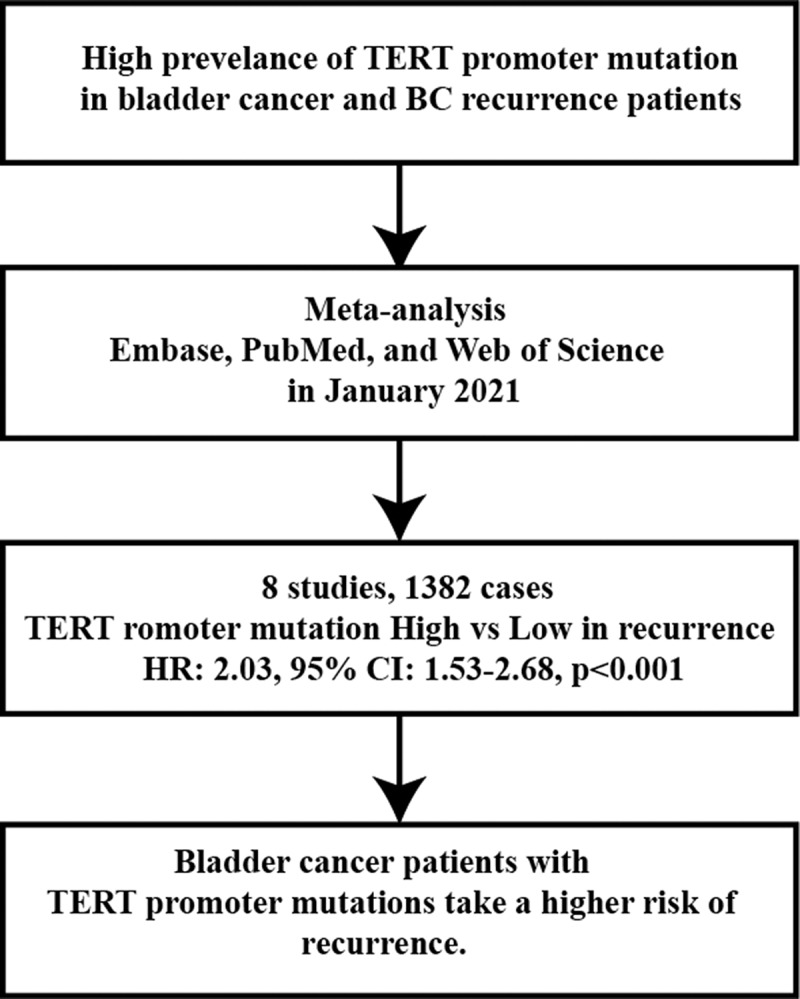

## Introduction

Telomeres are a kind of nucleoprotein complexes composed of many short non-transcribed DNA sequences, TTAGGG, proteins [1]. They are responsible for protecting the ends of chromosomes from being shorter as cells divide by reducing DNA sequences. Telomeres get shorter when cells divide. When the telomere length is short at a particular level, this cell will come to an end. Telomerase is a type of enzyme that can take telomere DNA TTAGGG into the end of chromosome to shorten telomere [2]. It is composed of telomerase reverse transcriptase (hTERT), telomerase RNA component (TERT), and telomerase associated protein 1 (htp1) [3]. Telomerase RNA component (TERC) plays a role as replication template, while TERT catalyzes the addition of DNA fragment TTAGGG [4]. Telomerase activity is low expressed in most normal and benign tumor tissues, but highly expressed in malignant tumors, which is associated with the ability of persistent division of malignant tumor cells [5]. Therefore, TERT mutation has been a crucial research hotspot in the pathogenesis of tumor since 2013 when it was firstly reported [6]. TERT gene locates in chromosome 5 and has 16 exons and 15 introns, with a total length of 35 KB [4]. The crucial promoter of TERT, located 330 bp in front of the translation initiation site, is one of the most commonly described mutation sites in studies. TERT promoter mutations mainly appeared in two hot spots (c250t and c228t) of 1,295,250 c > T and 12,952,228 c > T [5]. These two mutations are associated with the ATG (initiation codon) and the aggressiveness of tumors. Therefore, we hypothesized that TERT promoter mutation was associated with bladder cancer recurrence. We performed a meta-analysis to examine and summarize all data on TERT promoter mutations in bladder cancer and verified the above hypothesis.

## Methods

### Search strategy

**Figure 1.**
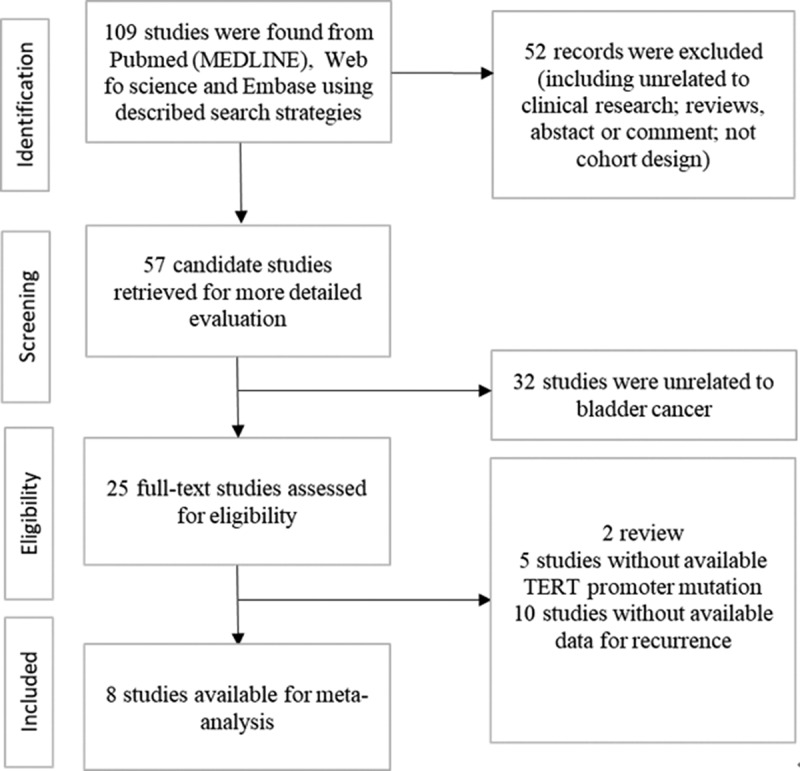
Schematic flow diagram for selection of included studies

This meta-analysis was performed according to PRISMA guidelines (Preferred Reporting Items for Systematic Reviews and Meta-Analyses) [7]. We finished the search in Embase, Web of Science, and PubMed (MEDLINE) up to January 2021, and finally included 10 studies. We included all studies for a total of 1382 cases. The filtering process is shown in Figure 1. The search contains the following keywords: ‘bladder cancer’, ‘urothelial cancer’, ‘TERT’, ‘C228T’, ‘C250T’, and ‘prognosis’. All studies were published in English. To identify all possible eligible studies, a manual search was conducted on references cited from each original study.

## Inclusion and exclusion criteria

Qualified studies should meet the following inclusion criteria: (1) definite diagnosis of bladder cancer by pathology, (2) without other previous cancer history, (3) reporting the relationship between TERT promoter mutation and recurrence-free survival (RFS) and/or overall survival (OS), (4) full-text studies containing sufficient and available data for calculating hazard ratios (HRs) and its ninety-five percent confidence intervals (CIs), (6) related clinicopathological data were available. The exclusion criteria were listed in the following: (1) unrelated topics or other types of articles, such as abstracts and reviews; (2) without data on associations between TERT promoter mutation and BC; and (3) data were collected only from studies of animal models or cell lines. The major determinants of recurrence are clinicopathological features according to the European Association of Urology [8].

## Data extraction and quality assessment

We collected the following data from the included study: 1, author's name, year of publication; 2, number of patients; 3, number and percentage of mutation; 4, HR and 95% CI. The Cochrane Prognostic Studies group [9] was measured to assess the methodological quality in Revman 5 (Cochrane Library Software, Oxford, UK).

### Statistical analyses

Stata software (Stata Corporation, College Station, version 12.0, TX, USA) carried out the analysis. The relationship between TERT promoter mutations and BC prognosis was tested by calculating hazard ratios (HRs) and its ninety-five percent CIs and analyzing survival data’s quantitative aggregation. When survival data were presented only in Kaplan Meier curves, the methods of Williamson [10], Parmar [11], and Tierney [12] for extracting HRs with 95.0% CIs were applied. Chi-squared tests and I^2^ statistics were performed to test the existing heterogeneity of the enrolled studies. Statistically significant differences in heterogeneity existed when P < 0.05 and I^2^ > 50.0%. Fixed-effects or random-effects models were performed to test the pooled HRs. To assess publication bias, the symmetry of funnel plots was evaluated visually; meanwhile, Egger’s and Begg’s tests were formally performed. When the visual asymmetry of funnel plots was found with P < 0.05, publication bias existed. Sensitivity analysis was carried out for evaluating the stability and reliability of the meta-analysis.

## Results

Relevant literature was collected as planned, and then evaluated and analyzed the relationship between TERT promoter mutation and the recurrence of bladder cancer.

### Literature search and study qualities

**Figure 2.**
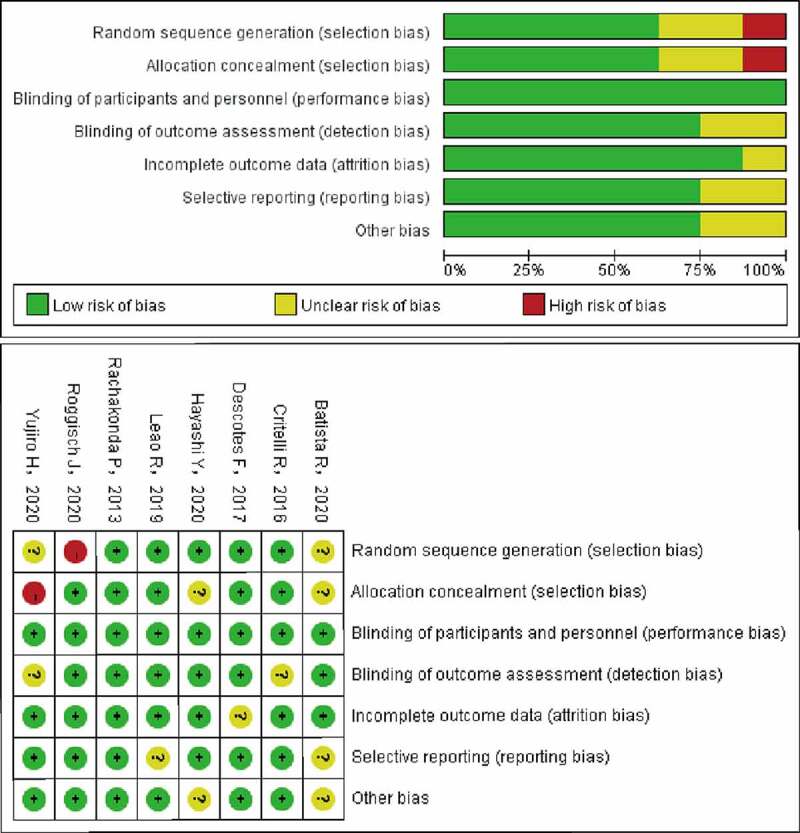
Methodological quality of the retrospective studies

Literature retrieval initially found 109 records, and 25 candidate studies were selected (Figure 1). After screening the full texts, 15 articles were excluded because there was no way to extract survival data from them. Ultimately, eight eligible studies [6,13–19] with available survival data were enrolled in this study. The main information and characteristics of the included studies are listed in Table 1. The enrolled studies, published between 2013 and 2020, were all conducted in patients with bladder cancer. One thousand three hundred and eighty-two cases with BC were enrolled in this study. The sample sizes for OS and RFS were 354 and 1382, respectively. One study did not mention mutation points [18] and the other one did not mention the follow-up time [20]. Figure 2 exhibits that the methodologic quality assessment of the enrolled studies was all good quality.

**Table 1.** Characteristics of the included studies

Study	Year	No. of patients	Percentage of mutation	Mutation point	Type of bladder cancer	Follow up time	Sample types
Roggisch, J [19]	2020	75	63 (84.0%)	C288T&C250T	urothelial	53	tissue
Hayashi, Y [17]	2020	54	16 (29.6%)	C288T&C250T	urothelial	60	tissue
Yujiro, H [16]	2020	35	6 (17.4%)	C288T&C250T	urothelial	72	urine
Batista, R [13]	2020	125	70 (56.0%)	C288T&C250T	urothelial	150	tissue
Leao, R [18]	2019	237	182 (76.8%)	not mentioned	urothelial	200	tissue
Descotes, F [15]	2017	348	280 (80.5%)	C288T&C250T	urothelial	60	urine
Critelli, R [14]	2016	229	119 (52.0%)	C288T&C250T	urothelial	96	urine
Rachakonda, P [6]	2013	279	186 (66.7%)	C288T&C250T	urothelial	180	tissue

### Association of TERT promoter mutations with recurrence of BC

**Figure 3.**
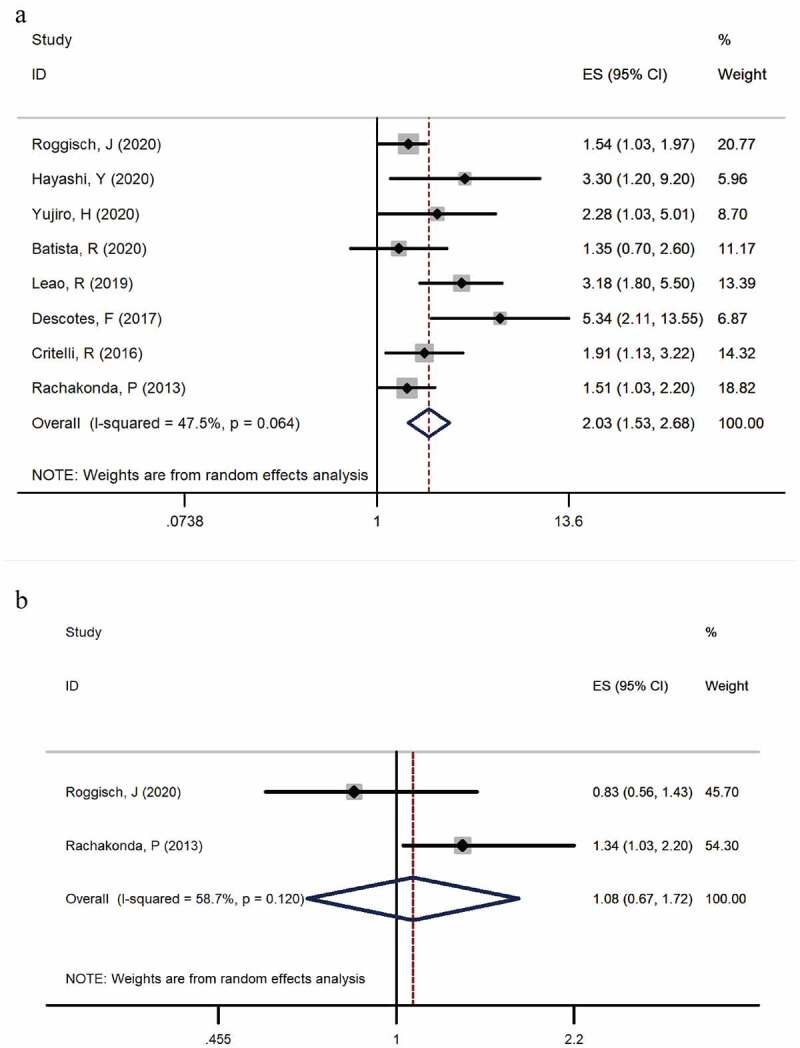
Forest plot of the HR analysis of TERT promoter mutation with RFS and OS of BC. A forest plot of RFS; B forest plot of OS

Eight studies [6,13–19] provided available data of recurrence in both mutation-positive and mutation-negative groups (heterogeneity: I^2^ = 47.5%, p = 0.064). Using pooled data, the results showed that TERT promoter mutation positive patients were more likely to relapse bladder cancer according to the meta-analysis (HR: 2.03, 95% CI: 1.53–2.68, p < 0.001, Figure 3a). In case number subgroup, the HR was 1.84 (95% CI, 1.25–2.71, p = 0.002) in less than 100 case subgroup and 2.13 (95% CI, 1.53–2.68, p < 0.001) in nonrandom subgroup, respectively. Three studies [14–16] examined TERT promoter mutations in urine, and five studies [6,13,17–19] examined TERT promoter mutations in bladder tissues. In specimen’s subgroup analysis, the HR was 2.03 (95% CI, 1.53–2.68, p < 0.001) in urine subgroup and 1.83 (95% CI, 1.33–2.51, p < 0.001) in tissue subgroup. Subgroup analysis results are exhibited in Figure 5 and Figure 6.

Association of TERT promoter mutations with overall survival of BC

Two studies [6,19] provided available data on overall survival in the TERT mutation-positive and TERT mutation-negative group (heterogeneity: I^2^ = 58.7%, p = 0.120). Both studies examined the mutation in tissues. In the pooled data, the results showed that patients with mutations took a higher risk of relapsed bladder cancer according to the meta-analysis (HR: 1.077, 95% CI: 0.674–1.718, p = 0.757, Figure 3b).

### Publication bias and sensitivity analysis

**Figure 4.**
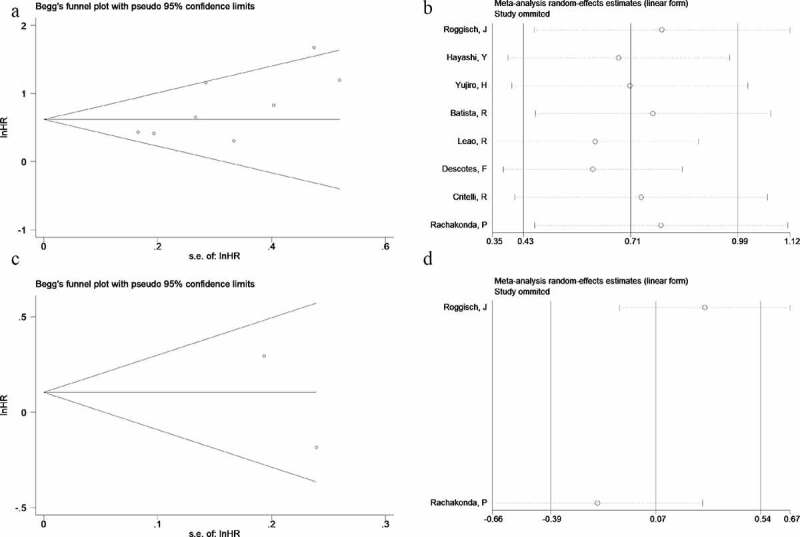
Begg’s funnel plots, and sensitivity analysis of the HR analysis of TERT promoter mutation with RFS and OS of BC. A funnel plot of RFS; B sensitivity analysis of RFS; C funnel plot of OS; D sensitivity analysis of OS

Egger’s test and Begg’s funnel plot were performed to evaluate the potential publication bias of enrolled studies in this meta-analysis. Significant publication bias was not detected for RFS groups (symmetrical shape of funnel plots and Begg’s test: P = 0.536, Egger’s test: P = 0.413, Figure 4a) or OS groups (symmetrical shape of funnel plots, Figure 4c). Testing the impact of each study on both RFS groups and OS groups’ pooled HR, and verify the results’ robustness, we performed sensitivity analysis by removing single study in sequence and obtained pooled HR for the remaining studies simultaneously. Sensitivity analysis showed that the influence of pooled HR was not significant when excluding any single study, which suggested that the results were relatively robust (Figure 4b, Figure 4d).

**Figure 5.**
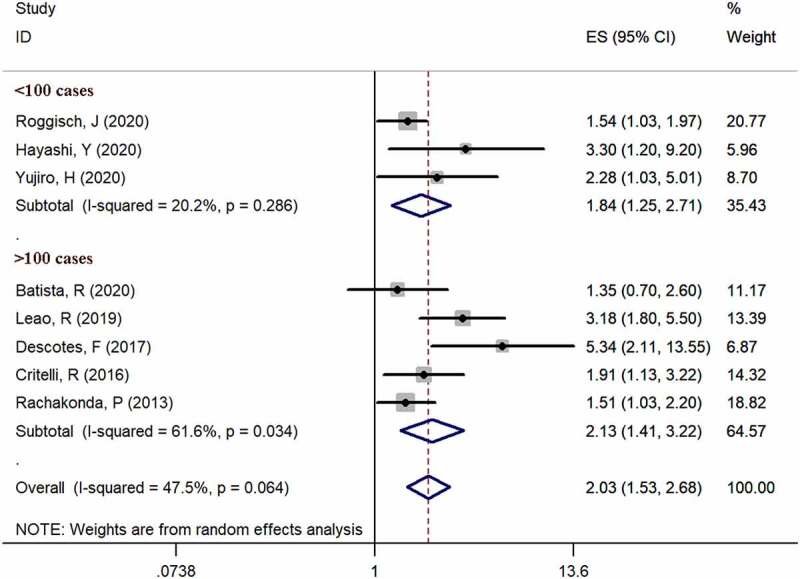
Case number subgroup analysis of the HR analysis of TERT promoter mutation with RFS of BC

**Figure 6.**
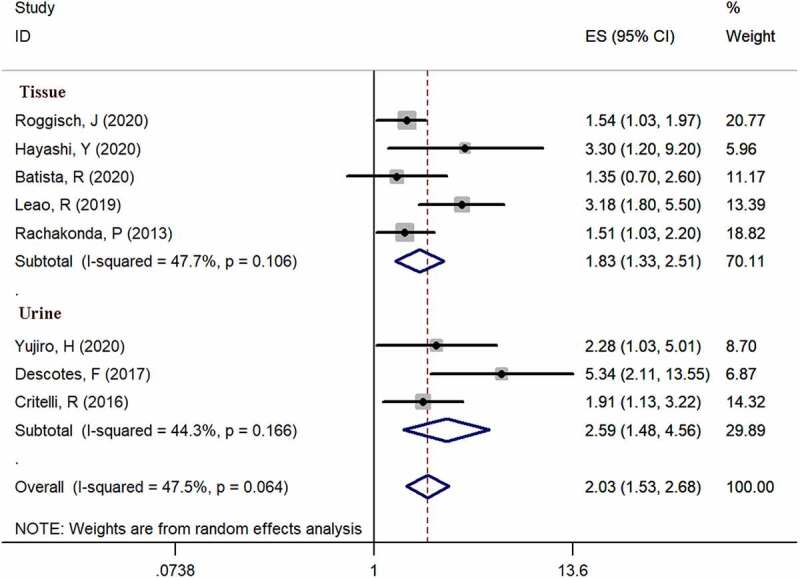
Specimen’s subgroup analysis of the HR analysis of TERT promoter mutation with RFS of BC

## Discussion

Bladder cancer is the sixth most prevalent cancer in the world, which the incidence rate has been increasing in recent years [21]. The increase in incidence rate, and the high cost of monitoring every patient, has brought a heavy burden to the public health system [22]. Prompt treatment, including complete transurethral resection, can make the five-year-long survival rate more than 90%. However, as high as 70–80% of the relapses make recurrence a major challenge for clinical management [23]. TERT promoter mutations were occurred in 30% to 84% of BC cases [6,13–20,24]. These results indicate that TERT promoter mutations are very frequent genomic events reported in BC. TERT promoter mutations can be helpful for early detection of recurrence and better adaptation to follow-up frequency and treatment. Many studies have reported that TERT promoter mutations were related to recurrence in various malignancies [25–28]. However, the potential of TERT promoter mutations’ prediction value in BC is still controversial. For example, Batista et al. [13] indicated that TERT promoter mutations did not significantly relate to recurrence of BC (HR: 1.352, 95% CI: 0.703–2.600, p = 0.367). Until now, no meta-analysis has been conducted to describe the prognostic value of TERT promoter mutations in BC patients. We produced this meta-analysis of eight studies including 1382 cases, to describe the prognostic value of TERT promoter mutations for recurrence in BC patients. This is the first meta-analysis to explore whether TERT promoter mutations made patients’ prognosis worse.

We found that the TERT promoter mutations were related to BC recurrence. The presence of mutations was not related to OS of BC. Mutations of the TERT promoter in BC resulted in increased TERT mRNA levels and increased tumor invasiveness [29]. The results were consistent with urine and tissue specimens. Many studies have shown that TERT promoter mutations significantly decreased RFS [15,18] and OS [24,30]. However, there were also contradictory ideas in this area. Allory [29] showed that OS was not associated with TERT promoter mutations. In contrast, there were studies of other tumors in which TERT promoter mutations were related to higher grade and reduced survival [31,32]. However, in order to predict the survival of patients with BC, it is necessary to find more than one biomarker.

The intertumoral molecular heterogeneity of BC made it hard to identify prognostics and biomarkers and targets for treatment or chemotherapy. The high frequency of occurrence of mutation made TERT the most common mutated gene in UBC. Therefore, it became a potential therapeutic target. Studies using strategies of telomerase inhibition have shown that strong TERT inhibition can result in progressive telomere shortening and ultimately to cancer cell apoptosis, including using of small-molecule inhibitors [33], immunotherapy [34] and antisense oligonucleotides [35]. At present, many anti-telomerase therapies are evaluated in clinical trials for many types of cancer, and brings hope for future treatment.

The limitations of this study were listed in the following. Firstly, all the enrolled studies were published in English, which may lead to publication bias. Second, the approaches for assessment of TERT promoter mutation were lack of uniform standard which might influence the results. Some studies received clinical samples from urine cytology, while others got from bladder biopsy. Furthermore, some data extracted from KM survival curves of included studies may be less reliable than data obtained directly. Lastly, many studies did not provide recurrence data of different types of mutations, respectively.

## Conclusion

Bladder cancer patients with TERT promoter mutations take a higher risk of recurrence. How these mutations affect the occurrence or development of bladder cancer has not been found. TERT promoter mutations may become a potential prediction factor for recurrence and a potential therapeutic target.

## Supplementary Material

Supplemental Material

## Highlights:

1. Telomerase reverse transcriptase (TERT) promoter mutations are common genetic event in bladder cancer.
2. TERT promoter mutations can be detected in both tissue and urine.
3. TERT promoter mutations can predict the recurrence of bladder cancer.
