# Supplementary material for: The role of telomerase reverse transcriptase (TERT) promoter mutations in prognosis in bladder cancer
Source: Bioengineered. 2021 May 2;12(1):1495–504. doi: 10.1080/21655979.2021.1915725 (PMC8806350; doi:10.1080/21655979.2021.1915725)
Supplement: Supplemental Material [file KBIE_A_1915725_SM6501.zip › supplement/Highlightsclean.docx]

Highlights:

1. Telomerase reverse transcriptase (TERT) promoter mutations are common genetic event in bladder cancer.
2. TERT promoter mutations can be detected in both tissue and urine.
3. TERT promoter mutations can predict the recurrence of bladder cancer.
