# Supplementary figures and images for: The role of telomerase reverse transcriptase (TERT) promoter mutations in prognosis in bladder cancer
Source: Bioengineered. 2021 May 2;12(1):1495–504. doi: 10.1080/21655979.2021.1915725 (PMC8806350; doi:10.1080/21655979.2021.1915725)

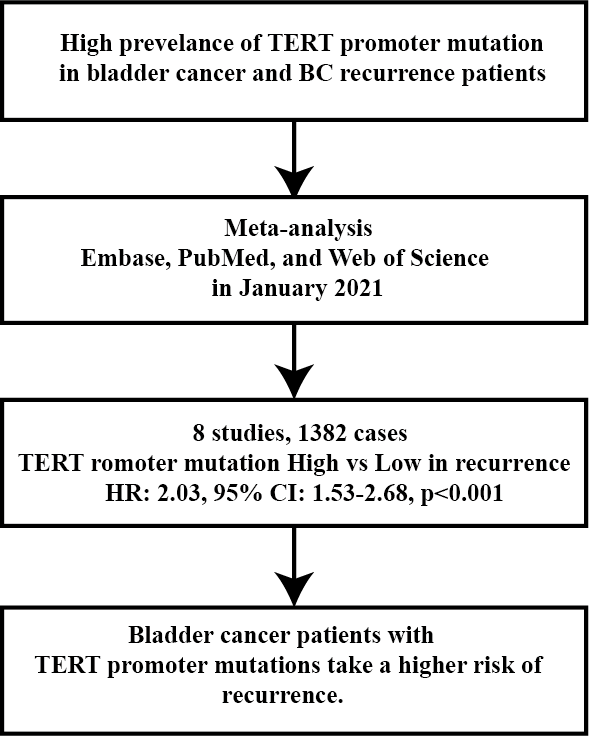

Supplement: Supplemental Material [file KBIE_A_1915725_SM6501.zip › supplement/Graphical Abstract.png]
